# Impact of the initial site of metastases on post-recurrence survival for neuroendocrine cervical cancer

**DOI:** 10.1186/s12885-022-09737-4

**Published:** 2022-06-14

**Authors:** Baoyue Pan, Ting Wan, Yinan Jiang, Xiaojing Zheng, Pingping Liu, Huiling Xiang, Min Zheng

**Affiliations:** 1 State Key Laboratory of Oncology in South China, Collaborative Innovation Center for Cancer Medicine, Sun Yat-Sen University Cancer Center, 651 Dongfeng Road East, Guangzhou, 510060 People’s Republic of China; 2 Department of Gynecology, Sun Yat-Sen University Cancer Center, 651 Dongfeng Road East, Guangzhou, 510060 People’s Republic of China

**Keywords:** Neuroendocrine cervical cancer, Abdominal organ recurrence, Prognosis, Immune therapy

## Abstract

**Objective:**

To summarize the risk factors and emphasize the prognostic importance of the site of recurrent neuroendocrine cervical cancer (NECC).

**Methods:**

We enrolled 88 patients who developed recurrence after radical surgery for pathological stage I–IVa primary NECC between January 2003 and 30 December 2020 and classified these cases into 7 groups based on the initial recurrence. The risk factors for post-recurrence survival (PRS) were analyzed by Kaplan–Meier and Cox regression methods.

**Results:**

Among 88 NECC patients, nearly all patients (95.50%) experienced progression within 3 years. The time to progression was significantly longer in patients with lung recurrence than in patients without lung recurrence (*p* = 0.008). After the first recurrence, the median follow-up was 11.1 months (range 2.37–65.50 months), and the 5-year PRS was only 20.6%. The depth of invasion in the primary surgery, number of recurrent sites, abdominal organ recurrence were correlated with PRS by univariate analysis**.** Multivariate analyses revealed that the number of recurrent sites (*p* = 0.025) and abdominal organ recurrence (*p* = 0.031) were independent prognostic factors. Notably, the combination of immune checkpoint inhibitors and chemotherapy, with or without surgery, showed a 43.8% objective response rate in recurrent NECC.

**Conclusion:**

Patients with abdominal organ recurrence need more sophisticated therapy. The combination of immune therapy and chemotherapy might be an opportunity for recurrent NECC.

**Supplementary Information:**

The online version contains supplementary material available at 10.1186/s12885-022-09737-4.

## Introduction

Neuroendocrine cervical cancer (NECC) is rare, accounting for less than 5% of all cervical tumors [1, 2]. According to the 2014 World Health Organization (WHO) classification of tumors of the female reproductive organs, NECC has two groups: carcinoid tumors and atypical carcinoid tumors are referred to as low-grade NECC, and small cell neuroendocrine cervical cancer (SCNECC) and large cell neuroendocrine cervical cancer (LCNECC) are high-grade NECC [3]. The 5-year survival rate of NECC is 37% for the early stages and 9% for the advanced stages [4]. More than 50% of NECC patients relapse within 5 years despite systemic therapies [5, 6], which is a much higher rate than that of conventional cervical tumors (20–40%) [7]. Given the aggressive nature of the NECC, it is important to identity the recurrence pattern and establish a risk model.

However, most studies on the recurrence pattern of NECC are case reports, and the patients collected in these studies are relatively limited to predicting prognosis [8–10]. In addition, few studies have reported the response to immune checkpoint inhibitors (ICI) therapy in NECC.

In conventional recurrent cervical tumors, lung metastasis indicates favorable clinical outcomes [11]. The number of distant metastatic sites is one of the independent factors for metastatic pancreatic neuroendocrine carcinoma [12]. With this background, we speculate that the prognosis of recurrent NECC is related to recurrent sites. We summarized the recurrent patterns of NECC in our center and discussed the prognostic factors in this study.

## Methods

### The selection of patients

We reviewed 126 patients who were diagnosed with recurrent NECC at Sun Yat-sen University Cancer Center from 1 January 2003 to 30 December 2020. Among them, 19 patients were followed up for less than 60 days after the first recurrence, 8 patients were at stage IVb according to the International Federation of Gynecology and Obstetrics (FIGO 2018) criteria, and 11 patients did not undergo radical hysterectomy. These 38 patients were excluded from the study. Finally, 88 patients with FIGO stages I–IVa were included in the study.

This retrospective study was performed in accordance with the Declaration of Helsinki and approved to waive informed patient consent by the institutional review board of Sun Yat-sen University Cancer Center (approval number: B2020-330–1) due to the observational and noninterventional study, and the patient’s data were kept under strict control. The authenticity of this article has been validated by uploading the key raw data onto the Research Data Deposit public platform (www.researchdata.org.cn), with the approval RDD number as RDDA2021001979.

### Baseline data collection

Demographic and clinicopathological data were collected from hospital records, including age, high-risk HPV human papillomavirus (HPV) test, FIGO stage, primary tumor size, pathology, and treatment when the patients were first diagnosed with NECC. When the tumor relapsed, data related to the pattern of recurrence and the follow-up treatment were collected.

### Diagnosis of recurrent NECC

The diagnosis of NECC was dependent on immunohistochemical analysis of the primary tumor or metastasis, which was demonstrated using several markers, including chromogranin A, synaptophysin, and CD56. Sometimes, neuron-specific enolase (NSE) was also used. Some patients were referred to as NECC not otherwise specified (NECCNOS) if they did not have a typical morphological classification but exhibited neuroendocrine markers. All available pathological slides were reviewed together by gynecological pathologists at Sun Yat-sen University Cancer Center.

To define recurrence, imaging examinations, such as F-fluoro-2-deoxy-d-glucose (FDG) positron emission tomography and CT (FDG-PET/CT), or contrast-enchanced computed tomography (CT), or magnetic resonance imaging (MRI), were employed as necessary investigations. A 20% increase in lesion size on a follow-up imaging scan within 3 months was also defined as recurrence when disease progressed in the primary treatment.

In this study, organ metastases that recurred for the first time after surgery were categorized into 7 initial recurrence sites: lung recurrence (*n* = 34), abdominal organ recurrence (*n* = 33), pelvic organ recurrence (*n* = 24), bone recurrence (*n* = 11), cervicothoracic lymph node recurrence (*n* = 10), brain recurrence (*n* = 7), and vaginal vault recurrence (*n* = 7). The effect of each initial recurrence site on postrecurrence prognosis was analyzed.

### Survival

Time to recurrence (TTR) was determined based on the time from the date of the diagnosis of NECC to the first recurrence by imaging evidence or histology. Post recurrence survival (PRS) was defined as the time from the date of the first diagnosis of recurrence to the follow-up deadline or the date of death.

### Immune checkpoint inhibitors (ICI) therapy

The ICI therapy was employed in 22 patients. 16 patients of them received at least 3 cycles and underwent imaging examinations to assess response. 4 patients underwent detection of ICI biomarkers, included PD-1 (1/16), PD-L1 staining (2/16) and tumor mutation burden (1/16). The remained 14 patients all progressed after anti-angiogenesis therapy or several cycles of second-line chemotherapies or local radiotherapy. Because multimodality therapy showed no benefit for patients, doctors introduced the ICI therapy and fully informed the patients of the tradeoffs.

### Statistical analysis

For comparisons of groups, the χ2-test and Fisher’s exact test were used where appropriate. All survival curves were plotted using the Kaplan–Meier method, and log-rank tests were carried out to assess survival differences between groups. Univariate and multivariable forward stepwise Cox regression models were used for OS analysis. A difference of 0.05 was considered significant. SPSS 25.0 (IBM Corp., Armonk NY, USA) was used for the statistical calculations.

## Results

### Baseline characteristics

The median age of the 88 patients who experienced recurrence was 46 years. Among the 43 patients who underwent HPV-based screening, 93.0% had HPV infection. According to the 2008 FIGO staging system, 50, 22, 0, and 2 patients had stage I, II, III and IVa disease, respectively. We restaged the patients using the 2018 FIGO system, which showed that 42, 9, 27, and 2 patients had stage I, II, III, and IVa disease, respectively. SCNECC was the most common pathological subtype (80.7%), and LCNECC and NECCNOS accounted for only 19.3% of all cases of recurrence in patients. Unexpectedly, nearly a quarter of patients (23/88) had mixed NECC, and mixed adenocarcinoma (AdC) was much more prevalent than mixed squamous carcinoma (SqC). Among all neuroendocrine differentiation markers, synaptophysin, chromogranin A, and CD56 were expressed in 94.0%, 80%, and 90.5% of patients, respectively. In addition, immunohistochemistry showed that 82.7% of 52 patients tested were positive for NSE.

All patients underwent radical surgery, and 29 patients (33.0%) showed pelvic lymph node metastasis (PLNM). In the primary treatment, all patients underwent chemotherapy and approximately 81.8% of them received at least four cycles. In addition, 60.2% patients received adjuvant radiation therapy. Other clinicopathological variables are listed in Table 1.

**Table 1:** Patient, tumor, initial treatment characteristics

Parameters	N	%
Age, mean (range)	46 years (24–67 years)	
Histology		
Small cell	71	80.7
Pure	48	54.5
Mixed with AdC	17	19.3
Mixed with SqC or adenosquamous carcinoma	6	6.8
Large cell	8	9.1
Pure	6	6.8
Mixed with small cell	2	2.3
Neuroendocrine, NOS	9	10.2
Pure	6	6.8
Mixed with AdC	3	3.4
FIGO stage (2018)		
I	42	47.7
II	9	10.2
III	27	30.7
IVa	2	2.3
N/A	8	9.1
Tumor size		
≤ 4.0 cm	41	46.6
> 4.0 cm	19	21.6
N/A	28	31.8
Depth of cervical stromal invasion		
< 1/3	22	25.0
≥ 1/3	57	64.8
N/A	9	10.2
lymphovascular space invasion		
Yes	55	62.5
No	12	13.6
N/A	21	23.9
Pelvic lymph node metastasis		
Yes	29	33.0
No	53	60.2
N/A	6	6.8
Primary treatment		
Radiation	53	60.2
Cycle of chemotherapy		
< 4	16	18.2
≥ 4	72	81.8

*N*: Number

### Distribution of recurrent sites

Hematogenous metastases, especially lung and abdominal organ recurrence, were the most common recurrent sites. There were 34 (38.6%) patients with lung recurrence, 33 (37.5%) patients with abdominal organ recurrence, 24 (27.3%) patients with pelvic organ recurrence, 11 (12.5%) patients with bone recurrence, 10 (11.4%) patients with cervicothoracic lymph node recurrence, 7 (8.0%) patients with brain recurrence and 7 (8.0%) patients with vaginal vault recurrence. Among the patients with abdominal organ recurrence, there were 25 patients with liver metastasis, 5 patients with abdominal lymph node metastasis, 2 patients with adrenal gland metastasis, 2 patients with pancreas metastasis, 1 patient with splenic metastasis and 1 patient with kidney metastasis. Approximately half of the patients had metastases in more than one organ at first recurrence. Detailed distributions of metastatic sites are shown in Table 2.

**Table 2:** The initial site of recurrence

**Initial recurrence sites**	N	%
Lung	34	38.6
Abdominal organs	33	37.5
Pelvic organs	24	27.3
Bone	11	12.5
Cervicothoracic lymph node	10	11.4
Brain	7	8.0
Vaginal vault	7	8.0
Other (breast, thyroid gland)	2	4.5

*N*: Number

### The impact of site-specific metastases on TTR and PRS

**Fig. 1:**
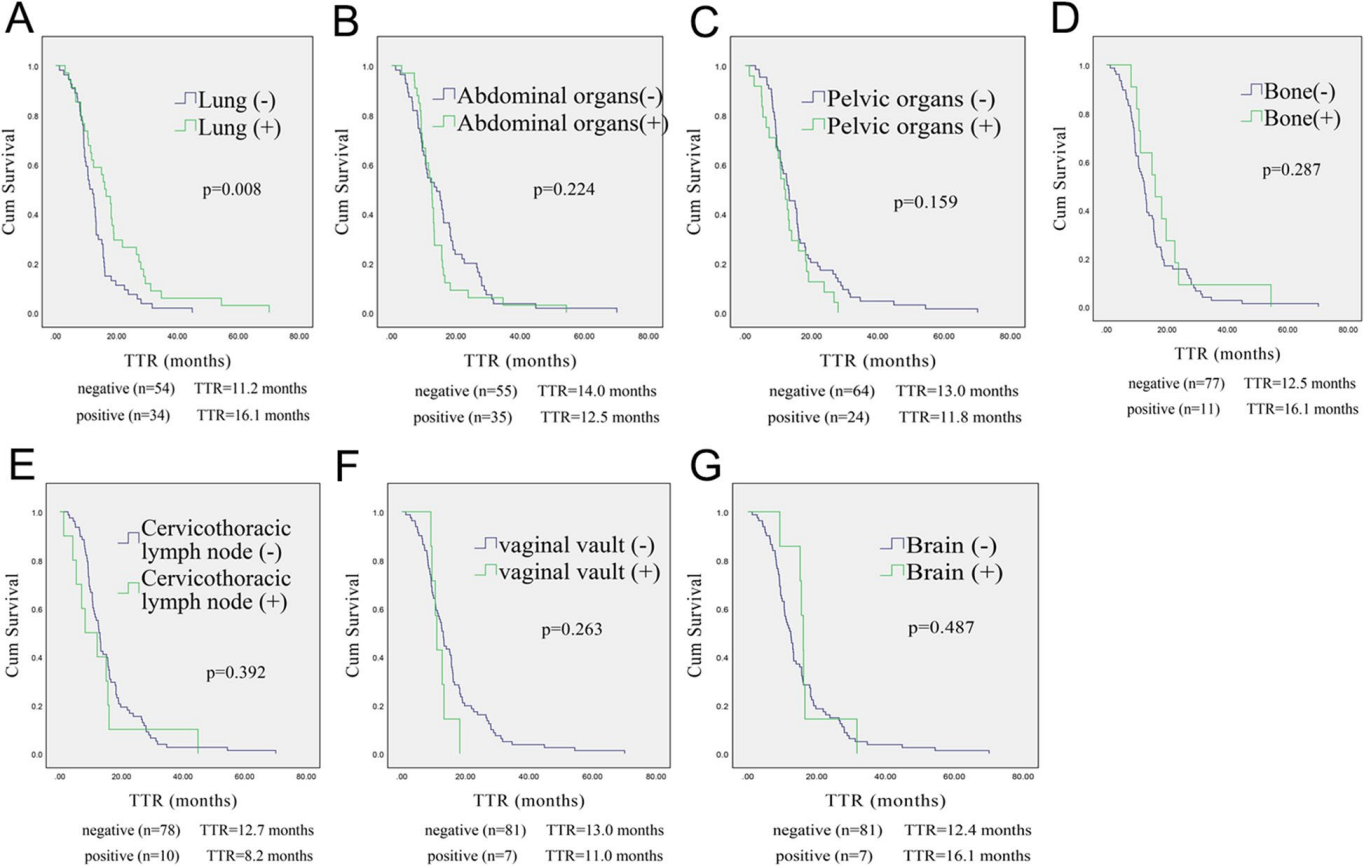
Kaplan–Meier curves of TTR for the 88 NECC patients according to different recurrence patterns. The TTR of patients with lung recurrence was better than that of patients without lung recurrence (**A**). There were no significant differences in the TTR of patients with or without recurrence at other sites (**B**-**G**). TTR: time to recurrence, NECC: neuroendocrine cervical cancer

Nearly all patients (95.50%) experienced postoperative progression within 3 years, and 99.86% of patients showed recurrence within 5 years. Only one patient showed recurrence in the 70th month. The TTR of patients with lung recurrence was better than that of patients without lung recurrence (16.1 months vs 11.2 months, *p* = 0.008) (Fig. 1A) Other recurrent sites have no effect on the TTR (Fig. 1 B-G).

**Fig. 2:**
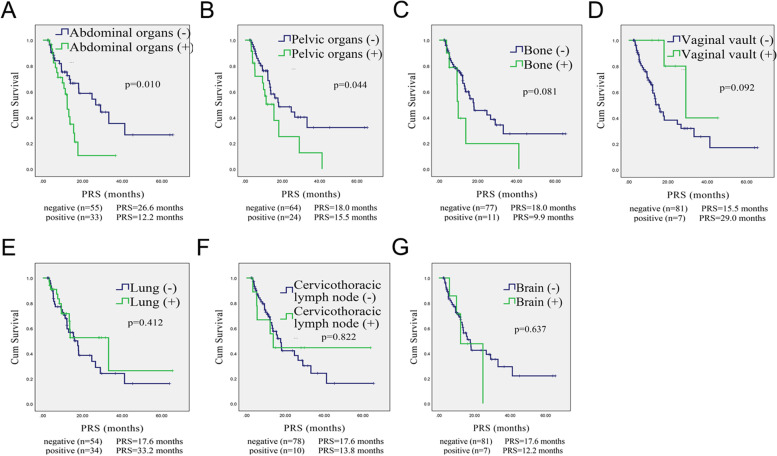
Kaplan–Meier curves of PRS for the 88 NECC patients according to different recurrence patterns. The PRS of patients with abdominal or pelvic organ recurrence was worse than that of the remaining patients (**A**-**B**). There were no significant differences in the RFS of patients with or without recurrence at other sites (**C**-**G**). PRS: post-recurrence survival, NECC: neuroendocrine cervical cancer

After the first recurrence, the median follow-up was 11.1 months (range 2.37–65.50 months), and the 5-year PRS was only 20.6%. The PRS of patients with abdominal or pelvic organ recurrence was worse than that of the remaining patients without abdominal or pelvic organ recurrence (12.2 *vs* 26.6 months, *p* = 0.010 and 18.0 *vs* 15.5 months, *p* = 0.044, respectively; Fig. 2A-B). The 3-year PRS was 10.4 and 66.5% among patients with or without abdominal recurrence, respectively. Other recurrent sites have no effect on the PRS (Fig. 2C-G).

We included the depth of invasion at the time of primary treatment and four recurrence-related factors (abdominal organs recurrence, pelvic organs recurrence, number of recurrent sites and ICI therapy) in the multivariate analysis. However, two or more recurrent sites and abdominal organ recurrence were finally correlated with worse outcome (Table 3).

**Table 3:** Univariate and multivariate analysis of PRS

	Univariate			Multivariate		
	*p*	HR	95% CI	*p*	HR	95% CI
Age (> 50 *vs*. ≤ 50)	0.662	0.859	0.436–1.695			
FIGO stage						
I + II *vs*. III + IV	0.121	1.653	0.875–3.124			
Histology						
NECC *vs.* Mixed NECC	0.571	0.818	0.408–1.641			
Tumor size (≤ 4 cm *vs*. > 4 cm)	0.770	1.125	0.511–2.476			
Invasion (Deep *vs*. shallow)	0.034	1.376	1.025–1.847			
LVSI	0.171	2.761	0.646–11.806			
Nerve invasion	0.178	1.803	0.765–4.249			
PLNM	0.229	1.406	0.739–2.677			
Syn	0.404	23.435	0.014–38,678.493			
cgA	0.504	1.297	0.606–2.777			
CD56	0.188	2.625	0.625–11.030			
Primary treatment						
Cycles of chemotherapy (< 4 *vs*. ≥ 4)	0.683	0.841	0.356–1.928			
Radiation	0.300	1.417	0.733–2.740			
Lung recurrence	0.412	0.753	0.382–1.484			
Abdominal organs recurrence	0.010	2.375	1.230–4.586	0.031	2.543	1.089–5.937
Pelvic organs recurrence	0.044	1.955	1.017–3.760			
Bone recurrence	0.081	2.088	0.914–4.775			
Cervicothoracic lymph node recurrence	0.822	0.897	0.350–2.303			
Brian recurrence	0.637	1.285	0.453–3.645			
Vaginal vault recurrence	0.092	0.293	0.070–1.222			
Number of recurrent sites (1 *vs*. > 1)	0.031	1.424	1.033–1.963	0.025	1.626	1.064–2.484
Treatment after recurrence						
Antiangiogenic therapy	0.234	0.483	0.146–1.601			
ICI therapy	0.079	0.342	0.103–1.133			

*LVSI* Lymphovascular space invasion, *PLNM* Pelvic lymph node metastasis, *Syn* Synaptophysin, *CgA* Chromogranin

Through further comparing patient characteristics with and without abdominal organ recurrence, we found that elderly patients (*p* = 0.022) and lung recurrence were observed less frequently in patients with abdominal organ recurrence than in those without abdominal organ recurrence (Table 4).

**Table 4:** Comparison of patient characteristic with and without abdominal organ recurrence

	Abdominal organs ( +) (*n* = 33)	Abdominal organs (-) (*n* = 55)	*p*
Age (> 50), n (%)	5 (15.2)	21 (38.2)	0.022
Histology (pure NECC), n (%)	23 (69.7)	39 (70.9)	0.904
FIGO stage (III + IV), n (%)	10 (33.3)	19 (38.0)	0.674
Tumor size (> 4 cm), n (%)	7 (31.8)	12 (31.5)	0.985
Invasion (Deep), n (%)	24 (80.0)	33 (67.3)	0.223
LVSI	25 (92.6)	30 (75%)	0.129
Nerve invasion	11 (50.0)	10 (27.8)	0.088
PLNM	10 (33.3)	19 (36.5)	0.770
Adjuvant radiation	22 (68.8)	31 (60.8)	0.462
Cycles of chemotherapy (≥ 4)	27 (81.2)	45 (83.3)	0.856
Number of recurrent sites (> 1), n (%)	19 (57.6)	20 (36.4)	0.052
Lung recurrence	8 (24.2)	26 (47.3)	0.032
Pelvic organs recurrence	8 (24.2)	16 (29.1)	0.621
Antiangiogenic therapy	5 (17.9)	9 (19.1)	0.890
ICI therapy	8 (28.6)	14 (29.8)	0.911

### Treatment of disease recurrence

We collected the detailed treatment of 75 patients after recurrence. Among these patients, 63 underwent chemotherapy, 26 received metastasis-directed radiation, and 20 received palliative surgery, transcatheter arterial chemoembolization (TACE), or radiofrequency ablation (RFA). However, antiangiogenic therapy (16/84) failed to improve survival until the follow-up deadline.

Among the 22 patients who received ICI therapy, 16 patients received at least 3 cycles, and the tumor response was assessed by imaging (Table 5). Based on the final tumor response, the objective response rate was 43.8% (7/16). However, among the rest 53 patients who received no PD-1 inhibitors, 41 patients were assessed by imaging and the objective response rate was only 22.0% (9/41).

**Table 5:** Response to PD-1 inhibitors in 16 patients

PD-1	Combined regimen	Best tumor response	Time to response (months)	Time to progression or the end of follow-up (months)	Initial recurrent site	Final disease status	Biomarker
Nivolumab	Paclitaxel + surgery	CR	-	7	Vaginal vault	PR	PD-L1 staining positively
Pembrolizumab	CPT11 + surgery	CR	4	8	Lung	CR	PD-1 staining positively
Camrelizumab	Paclitaxel + cisplatin	CR	4	5	Lung	CR	
Tislelizumab	Paclitaxel + cisplatin + surgery	CR	3	4	Lung	CR	
Sintilimab	Paclitaxel + cisplatin	SD	-	4	Lung	PR	
Pembrolizumab	Etoposide + cisplatin	PD	-	3	Lung	PD	
Toripalimab	Paclitaxel + cisplatin	PD	-	3	Lung	PD	
Nivolumab	Paclitaxel + cisplatin	SD	-	3	Liver	PD	
Sintilimab	Paclitaxel + cisplatin	PD	-	3	Abdominal organs	PD	
Tislelizumab/ Sintilimab	CPT11 + cisplatin/paclitaxel	PR	6	10	Pelvic organs	PD	
Sintilimab	Etoposide + cisplatin	PD	-	3	Abdominal organs + cervicothoracic lymph nodes	PD	
Pembrolizumab	CPT11	PR	2	14	Abdominal organs + Lung	PD	
Sintilimab	Paclitaxel	CR	6	12	Abdominal organs + pelvic organs + vaginal vault	CR	TMB-H
Tislelizumab	Paclitaxel	PR	3	3	Bone	PR	
Camrelizumab	CPT11 + ciaplatin	SD	-	4	Brian	SD	
Sintilimab	Paclitaxel + cisplatin	SD	-	3	Brian	SD	PD-L1 staining positively

The time to further progression or the end of follow-up after PD-1 treatment ranged from 3–14 months. The best tumor response to PD-1 inhibitors was as follows: progressive disease (4/16), stable disease (4/16), partial response (3/16) and complete response (5/16). All patients above received chemotherapy and three of them underwent surgery. The lung and vaginal vault were the most common initial recurrent sites of these patients who benefit from PD-1 treatment.

## Discussion

This study categorized the initial metastatic organs into 7 initial recurrence sites and demonstrated that the number of recurrent sites and abdominal organ recurrence were independent poor prognostic factors of PRS. However, other initial recurrence sites did not have any impact on the PRS.

Interestingly, our results showed that nearly one-third of patients were intermixed with AdC. Most cases of NECC have HPV infection (especially HPV 18), and some of them can be asymptomatic because of endophytic growth, which is common in cervical AdC [13]. Other neuroendocrine tumors are more similar to AdC than SqC. For example, in the lung and prostate, transdifferentiations from an AdC to neuroendocrine tumor occur in response to targeted therapy [14, 15]. However, in cervical cancer, the development of a neuroendocrine carcinoma from a small human papillomavirus–associated cervical adenocarcinoma has been reported lately [16]. Defining the molecular mechanisms of neuroendocrine transformation in cervical cancer remains a question.

Many studies have described the possible risk factors for primary NECC. FIGO stage has been proven to be a recognized factor [17]. In addition, tumor size, lymph node metastasis, chemotherapy cycles and other factors have different effects on survival [18]. However, the risk factors that affect prognosis after recurrence have not been investigated. In the present study, we conducted univariate and multivariate analyses, which indicated that the number of recurrent sites and abdominal organ recurrence were the main factors associated with PRS.

The common distant recurrent patterns in NECC are lung, abdominal organs, bone, and brain, which is the same as that of ordinary cervical cancer with hematogenous dissemination [19] and other sites of neuroendocrine tumors [20]. However, we found that lung metastasis had the highest rate among all recurrences in NECC, which is different from ordinary cervical cancer, whose pelvic relapse and distant lymphatic dissemination containing para-aortic lymph nodes or SCLN is relatively common [21, 22]. In particular, we revealed that lung metastasis in NECC occurred in the late postoperative period but had no effect on PRS. Only abdominal organ recurrence and pelvic organ recurrence were independent poor prognostic factors of PRS.

The most common primary treatment for NECC is radical surgery combined with chemotherapy in the early stage [23]. Chemotherapy regimens containing etoposide and platinum (EP) are recommended [24]. However, the treatment of recurrent disease is individualized and difficult, even in all cervical cancers [25, 26]. Most patients underwent chemotherapy containing platinum with etoposide or paclitaxel after recurrence. Some patients received palliative radiation and surgery. However, in the past decade, targeted therapy, such as anti-angiogenesis and immune therapy, has shown potential for treating resistant and recurrent cervical cancer [27–29]. Combinations comprising bevacizumab have been proven to improve the progression-free survival of recurrent SCNECC [30]. Pembrolizumab has also been used in recurrent neuroendocrine carcinoma of the lower genital tract, but when used alone, it shows minimal activity [31]. Only two case reports describe positive responses to nivolumab in NECC [10, 32]. Many patients with NECC received the latest therapy in this study. Regretfully, we obtained no significant results in multivariate analysis, likely due to the limited number of patients and short follow-up. In this study, three patients had PD-1/PD-L1 staining positively and one patient had high tumor burden, they all had disease controlled and three of them showed response to ICI, indicating the importance of biomarker detection [33]. Notably, lung and vaginal vault were slightly positively correlated with the response of PD-1 (*p* = 0.06) (Supplementary Table 1). In addition, a durable effect of PD-1 inhibitors was observed in two patients, similar to the effect in other tumors [34]. Both of them underwent biomarker detection of ICI therapy showed potential response to it. In addition, one of them had lung recurrence, which was more sensitive to PD-1 inhibitors than liver metastasis [35]. The other patient had the LCNECC histology, whose prognosis is better than that of SCNECC [8].

This retrospective study had inevitable limitations. Firstly, this was a single-center and small sample size study. In addition, selection biases also affected the prognosis of patients. Last but not least, only several patients had achieved genetic testing and not every patient underwent biomarker detection before ICI therapy. However, we collected as many patients as we could to focus on the pattern of recurrence and survival of patients with NECC. Our findings support the latest reports about targeted therapy and immune therapy and provide further insight into ICI therapy. Above all, we think that the pathogenesis and clinical manifestation of NECC are similar to those of cervical cancer. However, because of its special histology, the nature of this disease is more aggressive.

## Conclusion

Our results revealed that the number of recurrent sites and abdominal organ recurrence were significant prognostic factors in recurrent NECC. The combination of ICI and chemotherapy might be an opportunity for recurrent NECC on the basis of ICI biomarkers. International multicenter studies for recurrent NECC on various combinations of active chemotherapeutic agents, target therapies and immune therapy were warranted to improve the prognosis of recurrent NECC.

## Supplementary Information

**Additional file 1:** **Supplementary Table 1.** The site of recurrence and response of PD-1 inhibitors.

## Data Availability

The data that support the fundings of this study are available on request from the corresponding author. The data are not publicly available due to privacy or ethical restrictions.
