# Supplementary material for: Impact of the initial site of metastases on post-recurrence survival for neuroendocrine cervical cancer
Source: BMC Cancer. 2022 Jun 14;22:655. doi: 10.1186/s12885-022-09737-4 (PMC9195210; doi:10.1186/s12885-022-09737-4)
Supplement: Supplementary file 1 — Additional file 1: Supplementary Table 1. The site of recurrence and response of PD-1 inhibitors. [file 12885_2022_9737_MOESM1_ESM.docx]

**Supplementary Table 1. The site of recurrence and response of PD-1 inhibitors**

| Response  Recurrent sites | Stable disease and Progressive disease | Partial response and complete response | Total |
| --- | --- | --- | --- |
| Others | 6 | 1 | 7 |
| Lung and vaginal vault | 3 | 6 | 9 |
| Total | 9 | 7 | 16 |
